# Development of a nomogram for predicting in-hospital mortality in patients with liver cirrhosis and sepsis

**DOI:** 10.1038/s41598-024-60305-1

**Published:** 2024-04-29

**Authors:** Hai-rong Lin, Qiu-xia Liao, Xin-xin Lin, Ye Zhou, Jian-dong Lin, Xiong-jian Xiao

**Affiliations:** 1 Department of Intensive Care Unit, First Affiliated Hospital of Fujian Medical University, Fuzhou, 350004 China; 2 Department of Intensive Care Unit, National Regional Medical Center, Binhai Campus of the First Affiliated Hospital, Fujian Medical University, Fuzhou, 350212 China

**Keywords:** Liver cirrhosis, Sepsis, Nomogram, Prognosis, In-hospital mortality, Diseases, Medical research, Risk factors

## Abstract

In this study, we aimed to investigate the risk factors associated with in-hospital mortality in patients with cirrhosis and sepsis, establish and validate the nomogram. This retrospective study included patients diagnosed with liver cirrhosis and sepsis in the Medical Information Mart for Intensive Care IV (MIMIC-IV). Models were compared by the area under the curve (AUC), integrated discriminant improvement (IDI), net reclassification index (NRI) and decision curve analysis (DCA). A total of 1,696 patients with cirrhosis and sepsis were included in the final cohort. Our final model included the following 9 variables: age, heartrate, total bilirubin (TBIL), glucose, sodium, anion gap (AG), fungal infections, mechanical ventilation, and vasopressin. The nomogram were constructed based on these variables. The AUC values of the nomograms were 0.805 (95% CI 0.776–0.833), which provided significantly higher discrimination compared to that of SOFA score [0.684 (95% CI 0.647–0.720)], MELD-Na [0.672 (95% CI 0.636–0.709)] and ABIC [0.674(95% CI 0.638–0.710)]. We established the first nomogram for predicting in-hospital mortality in patients with liver cirrhosis and sepsis based on these factors. This nomogram can performs well and facilitates clinicians to identify people at high risk of in-hospital mortality.

## Introduction

Liver cirrhosis is a chronic progressive liver disease caused by various causes leading to the replacement of healthy liver parenchyma by fibrotic tissue and regenerative nodules^1^. Compared to patients without liver cirrhosis, patients with liver cirrhosis often have a 4–5 times higher risk of developing infections due to acquired immunodeficiency caused by hypersplenism resulting in leukopenia and lack of immune protein production^2–4^. In the occurrence of infection, patients with cirrhosis are at risk of developing sepsis and have a significantly higher mortality rate^4,5^. Therefore, the development of a scoring system for risk of death stratification to rapidly and accurately assess the prognosis of patients with cirrhosis and sepsis appears critical.

There are some scoring systems available for the prognostic assessment of patients with liver cirrhosis and sepsis respectively, such as the Sequential Organ Failure Scale (SOFA) score^6^, the Model for End-stage Liver Disease with the incorporation of serum sodium (MELD-Na)^7^, Age-Bilirubin-International Normalized Ratio (INR)-Creatinine (ABIC) Score^8^, etc. Due to the specificity of patients with liver cirrhosis and sepsis, these scoring systems have limited predictive value for the prognosis of these patients. However, there are few studies specifically focused on the prognosis of patients with liver cirrhosis and sepsis, and there are no predictive models used to predict in-hospital mortality in this group of patients. Thus, we conducted this study to identify important factors affecting the prognosis of patients with liver cirrhosis and sepsis from a large database, and to develop and validate a simple and effective nomogram for assessing the prognosis of patients with liver cirrhosis and sepsis to help clinicians to risk-stratify the prognosis of them and to develop individualized treatment strategies.

## Materials and methods

### Data sources

This is a retrospective study from the Medical Information Mart for Intensive Care IV (MIMIC-IV, version 2.2), a large, open-access, single-center database^9^. The database covers de-identified health records of patients admitted by the intensive care unit (ICU) of Beth Israel Deaconess Medical Center (BIDMC, Boston, Massachusetts, USA) from 2008 to 2019. These records include admission records, International Classification of Diseases 9th edition (ICD-9) or ICD-10 diagnoses, demographics, survival information, vital signs, laboratory tests, medication records, fluid balance, microbiological culture results, caregiver records, and other details. All patient information in the database is anonymous and therefore does not require informed consent. One author (HR Lin) has completed the CITI examination and received permission to use the database for research purposes (certification number: 11383789).

### Participants

**Figure 1:**
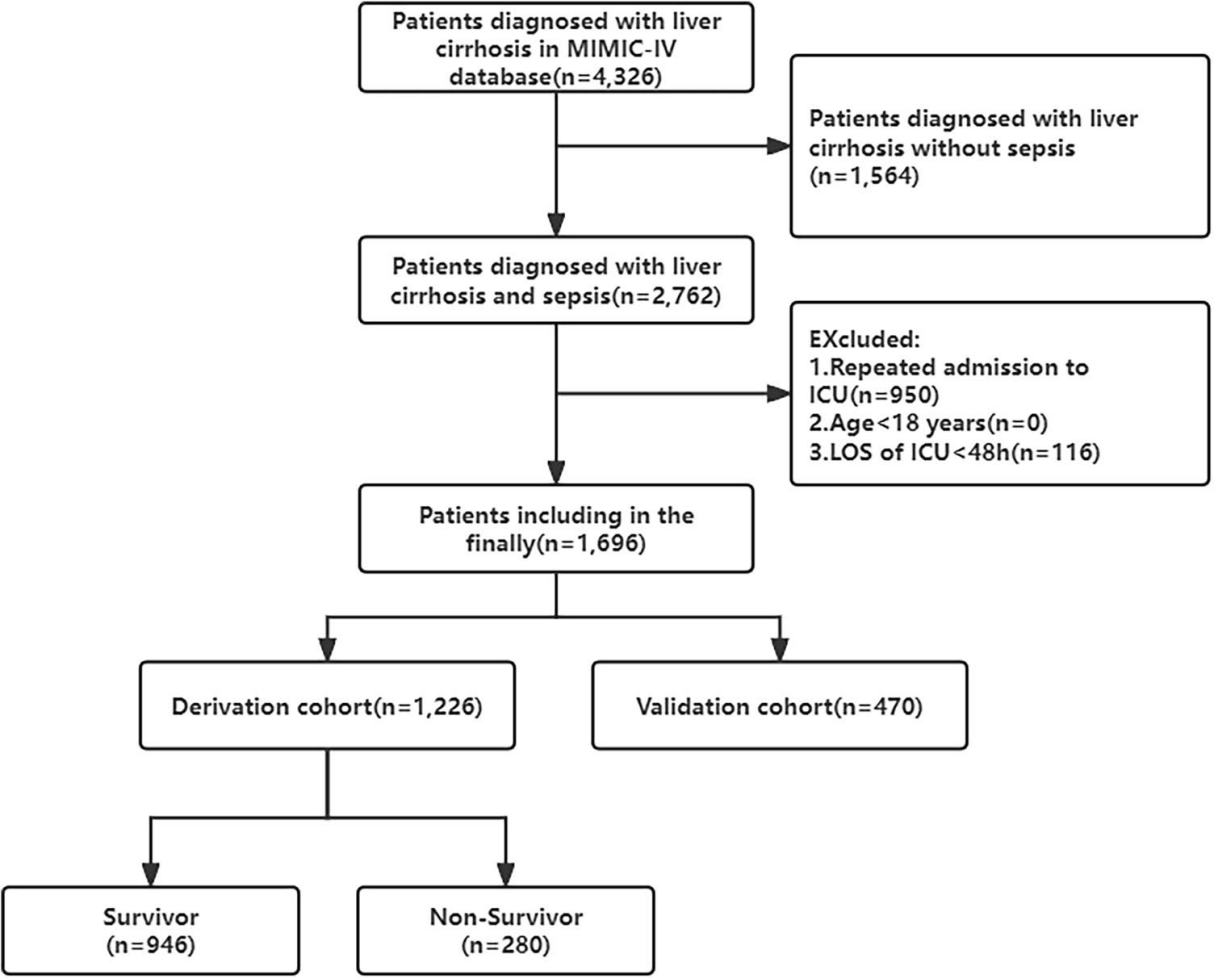
The flowchart of patient selection. *MIMIC-IV* the medical information mart for intensive care IV, *ICU* intensive care unit, *LOS* length of stay.

Our study included patients with a diagnosis of liver cirrhosis identified by ICD-9 or ICD-10 codes. Patients with suspected infection and a SOFA score of ≥ 2 were also considered as sepsis according to the definition of sepsis 3.0^10^. Patients with multiple ICU admissions had only the first hospitalization data extracted. Patients aged < 18 or length of stay (LOS) of ICU < 48 h were excluded from the study. The screening process is shown in Fig. 1.

### Data extraction

We used Structured Query Language (SQL) via Navicat Premium (version 15.0.12) to extract data from the MIMIC-IV database, including demographic characteristics, hospitalization information, survival information, complications, causes of liver cirrhosis, comorbidities, vital signs, laboratory data, continuous renal replacement therapy (CRRT), mechanical ventilation, vasopressin, albumin use within 48 h of ICU admission, Infections position, microbiological culture results and severity of illness score, etc. Demographic information included patient gender, age, marital status, and race. Complications included hypertension, diabetes, chronic heart failure (CHF), chronic obstructive pulmonary disease (COPD), and rheumatic disease. Causes of liver cirrhosis include alcoholic, viral, and biliary. Comorbidities include hepatic encephalopathy (HE), esophageal varices with hemorrhage (EVH), liver failure, septic shock, etc. Vital signs included heartrate (HR), mean blood pressure (MBP), and respiratory rate (RR) at the time of ICU admission. Laboratory parameters were those first obtained on admission to the ICU and included white blood cells (WBC), hemoglobin (HGB), mean red blood cell hemoglobin (MCH), mean red blood cell volume (MCV), red blood cell distribution width (RDW), platelets(PLT), total bilirubin (TBIL), albumin (ALB), alanine aminotransferase (ALT), lactate dehydrogenase (LDH), serum creatinine (Scr), sodium, bicarbonate, Anion gap (AG), prothrombin time (PT), and INR. Severity scoring systems included SOFA score, MELD-Na, and ABIC score. Microbiological culture results included bacterial infections, fungal infections, and bacterial combined with fungal infections.

### Statistical analysis

Data were integrated using Stata (version 17.0, Texas, USA) to obtain a complete table of hospitalization information for patients with liver cirrhosis and sepsis. To minimize bias due to missing data, variables with more than 20% missing values (e.g., lactate, uric acid, etc.) were excluded from the final cohort. Missing values for continuous variables were replaced using the median^11^. There were no missing values for all categorical variables. R software (V.4.2.3. https://www.r-project.org/) was used for statistical analysis. The Shapiro–Wilk test was used to confirm whether the continuous variables conformed to a normal distribution. Continuous variables conforming to a normal distribution were described using the mean and standard deviation values. Non-normality continuous variables were described using median and interquartile range (IQR). Differences between groups were identified using t-test or Wilcoxon rank sum test, respectively. Categorical variables were used for description using frequencies and percentages, and chi-square tests were used to identify differences between groups. The least absolute shrinkage and selection operator (LASSO) logistic regression was used to screen potential candidates. When the cross-validation error was within one standard error of the minimum, the maximum value of lambda was selected to screen candidates. Subsequently, multivariate logistic regression analysis was performed based on the screened variables from the LASSO regression, where variables with P < 0.05 were included in the final model, and the results were expressed as ratio (OR) and 95% confidence interval (CI). Ultimately, nomogram were developed based on the final multivariate analysis model. The area under the receiver operating characteristic curve (AUC), sensitivity, specificity, integrated discriminant improvement (IDI), and net reclassification index (NRI) were calculated to assess the apparent performance of the nomograms compared to SOFA score, MELD-Na and ABIC score. Calibration curves were used to confirm the calibration performance of the nomogram model. The Hosmer–Lemeshow goodness-of-fit test (HL test) was calculated to compare the accuracy of the nomogram. Decision curve analysis (DCA) was plotted to assess the clinical usefulness of the prediction models. p < 0.05 was considered statistically significant.

## Results

### Baseline characteristics

As shown in Fig. 1, we screened a total of 4326 patients with a diagnosis of liver cirrhosis, resulting in 1696 adult patients enrolled in this study. The overall in-hospital mortality rate for patients with liver cirrhosis and sepsis was 23.8%. All patients were randomized into a derivation cohort (70%, n = 1226) and a validation cohort (30%, n = 470). The derivation cohort was used to create the nomograms, while the validation cohort was used to perform the validation. The number of in-hospital mortality occurred in the derivation and validation cohorts was 220 (22.8%) and 123 (26.2%), respectively. Table 1 shows the baseline characteristics between those who died and survived during hospitalisation, and between the derivation cohort and the validation cohort. The median age of patients who died in hospital was 60.2 years (IQR 53.0–67.4 years), which was significantly higher than that of patients who survived hospitalization (62.2 years, IQR 54.0–70.8 years). The majority of patients with liver cirrhosis and sepsis were male (65.7% and 66.0% in the alive and dead cohorts, respectively), more than female (34.3% and 34.0%). More patients who died during their hospital stay had CHF, HE, hepatic failure, and Septic shock than those who survived hospitalization. Surviving patients they were less likely to have infections in the blood, lung, abdomen, and urinary than those who died. Fungal infections and bacterial infections occur more commonly in patients who died in the hospital. Patients who died had lower MCHC, glucose, sodium, and bicarbonate values. However, WBC, MCH, MCV, RDW, TBIL, Scr, AG, PT, and INR levels in patients who died during their hospital stay were significantly increased. Additionally, the deceased patients received more CRRT, Mechanical ventilation, norepinephrine, vasopressin, carbapenem antibiotic, and albumin use in 48 h than the surviving patients. All variables were evenly distributed in the derivation and validation cohorts, except RDW and TBIL.

**Table 1:** Baseline characteristics of the patients with liver cirrhosis and sepsis.

Variables	Total (n = 1696)	Alive (n = 1293)	Dead (n = 403)	*p*-value for alive vs dead	Derivation cohort (n = 1226)	Validation cohort (n = 470)	*p*-value for derivation vs validation
Demographic variables							
Age, (years)	60.6 (53.3, 68.2)	60.2 (53.0, 67.4)	62.2 (54.0, 70.8)	0.004	60.4 (53.2, 68.0)	60.7 (54.2, 68.7)	0.527
Gender, n (%)							
Male	1115 (65.7)	849 (65.7)	266 (66.0)	0.947	819 (66.8)	296 (63.0)	0.153
Female	581 (34.3)	444 (34.3)	137 (34.0)		407 (33.2)	174 (37.0)	
Marital status, n (%)							
Married	693 (40.9)	533 (41.2)	160 (39.7)	0.628	485 (49.6)	208 (44.3)	0.088
Other	1003 (59.1)	760 (58.8)	243 (60.3)		741 (60.4)	262 (55.7)	
Race, n (%)							
White	1123 (66.2)	870 (67.3)	253 (62.8)	0.271	824 (67.2)	299 (63.6)	0.510
Black	132 (7.8)	102 (7.8)	30 (7.4)		94 (7.7)	38 (8.1)	
Asian	48 (2.8)	35 (2.7)	13 (3.2)		35 (2.9)	13 (2.8)	
Other	393 (23.2)	286 (22.1)	107 (26.5)		273 (22.3)	120 (25.5)	
Admission location, n (%)							
Emergency	829 (49.9)	642 (49.7)	187 (46.4)	0.279	608 (49.6)	221 (47.0)	0.371
Other	867 (51.1)	651 (50.3)	216 (53.6)		618 (50.4)	249 (53.0)	
First care unit, n (%)							
MICU/SICU	1403 (82.7)	1079 (83.4)	324 (80.4)	0.341	1021 (83.3)	382 (81.3)	0.403
CCU/CVICU	158 (9.3)	114 (8.8)	44 (10.9)		107 (8.7)	51 (10.9)	
Other	135 (8.0)	100 (7.7)	35 (8.7)		98 (8.0)	37 (7.9)	
Vital signs							
HR, (beats/min)	104.0 (91.0, 118.0)	102 (90.0, 116.0)	111.0 (94.5, 124.0)	< 0.001	104.0 (910, 118.0)	103.5 (80.6, 118.0)	0.311
MBP, (mmHg)	57.0 (50.0, 81.0)	58.0 (51.0, 83.0)	54.0 (48.0, 61.0)	< 0.001	58.0 (51.0, 81.5)	57.0 (50.0, 80.9)	0.643
RR, (times/min)	26.0 (23.0, 31.0)	26.0 (18.5, 30.0)	28.0 (24.0, 33.0)	< 0.001	26.0 (23.0, 31.0)	26.0 (17.6, 31.0)	0.230
Complications							
Hypertension, n (%)	541 (31.9)	430 (33.3)	111 (27.5)	0.037	384 (31.3)	157 (33.4)	0.444
Diabetes, n (%)	540 (31.8)	408 (31.6)	132 (32.8)	0.696	397 (32.4)	143 (30.4)	0.474
CHF, n (%)	331 (19.5)	227 (17.6)	104 (25.8)	< 0.001	237 (19.3)	94 (20.0)	0.808
COPD, n (%)	400 (23.6)	308 (23.8)	92 (22.8)	0.732	280 (22.8)	120 (25.5)	0.269
Rheumatic disease, n (%)	32 (1.9)	28 (2.2)	4 (1.0)	0.193	25 (2.0)	7 (1.5)	0.585
Renal disease, n (%)	380 (22.4)	275 (21.3)	105 (26.1)	0.052	270 (22.0)	110 (23.4)	0.585
Causes of liver cirrhosis							
Alcohol, n (%)	740 (43.6)	562 (43.5)	178 (44.2)	0.848	535 (43.6)	205 (43.6)	1.000
Viral, n (%)	427 (25.2)	333 (25.8)	94 (23.3)	0.360	311 (25.4)	116 (24.7)	0.819
Biliary, n (%)	43 (2.5)	36 (2.8)	7 (1.7)	0.324	28 (2.3)	15 (3.2)	0.373
HE, n (%)	364 (21.5)	252 (19.5)	112 (27.8)	< 0.001	261 (21.3)	103 (21.9)	0.830
EVH, n (%)	146 (8.6)	117 (9.0)	29 (7.2)	0.291	113 (9.2)	33 (7.0)	0.178
Hepatic failure, n (%)	204 (12.0)	131 (10.1)	73 (18.1)	< 0.001	155 (12.6)	49 (10.4)	0.241
Septic shock, n (%)	370 (21.8)	201 (15.5)	169 (41.9)	< 0.001	269 (21.9)	101 (21.5)	0.892
Laboratory test							
WBC, (K/UL)	9.2 (5.9, 14.6)	8.5 (5.5, 13.4)	11.6 (7.4, 17.6)	< 0.001	9.10 (5.9, 14.6)	9.5 (5.8, 14.6)	0.599
HGB, (g/L)	97.0 (83.0, 110.0)	9.7 (8.4, 11.0)	9.6 (8.1, 11.0)	0.288	96.0 (83.0, 110.0)	97.0 (82.0, 111.0)	0.737
MCH, (pg)	31.4 (29.4, 33.5)	31.3 (29.3, 33.2)	31.9 (29.9, 34.5)	< 0.001	31.4 (29.4, 33.3)	31.55 (29.4, 33.7)	0.306
MCHC, (%)	33.1 ± 1.8	33.2 ± 1.8	33.0 ± 1.7	0.035	33.1 ± 1.8	33.1 ± 1.7	0.632
MCV, (fL)	94.0 (89.0, 100.0)	94.0 (89.0, 99.0)	97.0 (91.0, 103.0)	< 0.001	94.0 (89.0, 100.0)	95.0 (90.0, 101.0)	0.106
RDW, (%)	16.7 (15.1, 18.7)	16.4 (14.9, 18.5)	17.2 (15.4, 19.5)	< 0.001	16.5 (14.9, 18.6)	17.0 (15.4, 19.1)	0.010
PLT, (K/UL)	103.0 (67.0, 155.3)	102.0 (67.0, 150.0)	107.0 (69.0, 179.5)	0.117	104.0 (69.0, 155.0)	101.0 (66.0, 157.0)	0.609
TBIL, (umol/L)	39.3 (22.2, 88.9)	37.6 (20.5, 75.2)	65.0 (30.0, 170.1)	< 0.001	37.6 (20.5, 83.8)	34.2 (22.2, 107.7)	0.013
ALB, (g/L)	30.0 (25.0, 32.0)	30.0 (26.0, 32.0)	30.0 (24.0, 32.5)	0.076	30.0 (25.0, 32.0)	30.0 (25.0, 33.0)	0.570
ALT, (IU/L)	35.0 (21.0, 72.0)	35.0 (21.0, 72.0)	35.0 (22.0, 71.0)	0.953	35.0 (21.0, 72.0)	35.0 (23.0, 72.0)	0.199
Scr, (umol/L)	106.1 (70.7, 185.6)	88.4 (61.9, 150.3)	159.1 (88.4, 256.4)	< 0.001	97.2 (70.7, 185.6)	106.0 (70.7, 185.6)	0.664
Glucose, (mg/dL)	126.0 (103.0,167.0)	127.0 (105.0, 169.0)	121.0 (95.0, 157.5)	< 0.001	124.0 (102.0,163.0)	129.0 (105.0,174.0)	0.085
Sodium, (mmol/L)	138.0 (133.0, 141.0)	138.0 (134.0, 141.0)	136.0 (132.0, 140.0)	< 0.001	138.0 (133.0, 141.0)	138.0 (134.0, 141.0)	0.532
Bicarbonate, (mEq/L)	22.0 (19.0, 24.0)	22.0 (19.0, 25.0)	20.0 (17.0, 24.0)	< 0.001	22.0 (19.0, 24.0)	21.0 (19.0, 24.0)	0.285
AG, (mmol/L)	15.0 (12.0, 18.0)	14.0 (12.0, 17.0)	17.0 (14.0, 21.0)	< 0.001	15.0 (12.0, 18.0)	15.0 (12.0, 18.0)	0.681
PT, (s)	17.7 (15.1, 22.0)	17.0 (14.7, 20.6)	20.9 (17.0, 27.6)	< 0.001	17.5 (15.0, 21.7)	18.0 (15.2, 22.2)	0.157
INR	1.6 (1.4, 2.0)	1.5 (1.3, 1.9)	1.9 (1.5, 2.6)	< 0.001	1.6 (1.4, 2.0)	1.7 (1.4, 2.0)	0.170
Infections position							
Blood, n (%)	190 (11.2)	111 (8.6)	79 (19.6)	< 0.001	131 (10.7)	59 (12.6)	0.275
Lung, n (%)	318 (18.8)	194 (15.0)	124 (30.8)	< 0.001	236 (19.2)	82 (17.4)	0.395
Abdomen, n (%)	304 (17.9)	212 (16.4)	92 (22.8)	0.004	222 (18.1)	82 (17.4)	0.751
Urinary, n (%)	314 (18.5)	213 (16.5)	101 (25.1)	< 0.001	227 (18.5)	87 (18.5)	0.998
Skin, n (%)	2 (0.1)	1 (0.1)	1 (0.2)	0.419	2 (0.2)	0 (0.0)	0.381
Microculture and Antibacterial agents							
Fungal infections, n (%)	217 (12.8)	118 (9.1)	99 (24.6)	< 0.001	152 (12.4)	65 (13.8)	0.429
Bacterial infections, n (%)	728 (42.9)	511 (39.5)	217 (53.8)	< 0.001	526 (42.9)	202 (43.0)	0.978
Bacterial and fungal infections, (%)	157 (9.3)	88 (6.8)	69 (17.1)	< 0.001	110 (9.0)	47 (10.0)	0.513
Carbapenem antibiotic, n (%)	160 (9.4)	79 (6.1)	81 (20.1)	< 0.001	113 (9.2)	47 (10.0)	0.621
β lactam antibiotic, n (%)	1439 (84.8)	1085 (83.9)	354 (87.8)	0.066	1042 (85.0)	397 (84.5)	0.788
Interventions							
Urinary catheter, n (%)	346 (20.4)	239 (18.5)	107 (26.6)	< 0.001	248 (20.2)	98 (20.9)	0.828
CRRT, n (%)	205 (12.1)	92 (7.1)	113 (28.0)	< 0.001	143 (11.7)	62 (13.2)	0.435
Mechanical ventilation, n (%)	563 (33.2)	366 (28.3)	197 (48.9)	< 0.001	397 (32.4)	166 (35.3)	0.275
Norepinephrine, n (%)	609 (35.9)	358 (27.7)	251 (62.3)	< 0.001	432 (35.2)	177 (37.7)	0.352
Vasopressin, n (%)	254 (15.0)	99 (7.7)	155 (38.5)	< 0.001	184 (15.0)	70 (14.9)	1.000
Terlipressin, n (%)	2 (0.1)	1 (0.1)	1 (0.2)	0.419	2 (0.2)	0 (0.0)	0.381
48 h-albumin use, n (%)	614 (36.2)	414 (32.0)	200 (49.6)	< 0.001	450 (36.7)	164 (34.9)	0.523
Scoring systems							
SOFA	8.0 (5.0, 10.0)	7.0 (5.0, 10.0)	10.0 (7.0, 13.0)	< 0.001	8.0 (5.0, 10.0)	8.0 (5.0, 11.0)	0.312
MELD-Na	22.2 (11.2, 33.6)	18.4 (10.4, 30.3)	29.8 (18.4, 41.9)	< 0.001	20.8 (11.2, 33.6)	22.0 (11.2, 33.5)	0.710
ABIC	11.6 (9.7,15.5)	11.2 (9.4, 14.2)	14.2 (11.0, 22.9)	< 0.001	11.5 (9.70,14.9)	11.9 (9.9,16.5)	0.516
LOS of hospital, (day)	9.9 (5.8, 17.7)	9.7 (5.8, 17.8)	10.7 (5.7, 17.0)	0.851	10.0 (5.8, 17.9)	9.7 (5.7, 17.3)	0.657
LOS of ICU, (day)	3.1 (1.8, 6.4)	2.7 (1.6, 5.2)	4.9 (2.8, 9.1)	< 0.001	3.1 (1.8, 6.4)	3.0 (1.8, 6.1)	0.429
In-hospital mortality, n (%)	403 (23.8)	–	–	–	280 (22.8)	123 (26.2)	0.168

*MICU* medical intensive care unit, *SICU* surgical intensive care unit, *CCU* coronary care unit, *CVICU* cardiovascular intensive care unit, *HR* heart rate, *MBP* mean blood pressure, *RR* respiratory rate, *CHF* chronic heart failure, *COPD* chronic obstructive pulmonary disease, *HE* hepatic encephalopathy, *EVH* esophageal varices with hemorrhage, *WBC* white blood cells, *HGB* hemoglobin, *MCH* mean red blood cell hemoglobin, *MCV* mean red blood cell volume, *RDW* red blood cell distribution width, *PLT* platelets, *TBIL* total bilirubin, *ALB* albumin, *ALT* alanine aminotransferase, *LDH* lactate dehydrogenase, *Scr* serum creatinine, *AG* anion gap, *PT* prothrombin time, *INR* international normalized ratio, *SOFA* the sequential organ failure assessment, *MELD-Na* the model for end-stage liver disease with the incorporation of serum sodium, *ABIC* age-bilirubin-INR-creatinine score, *CRRT* continuous renal replacement therapy, *LOS* length of stay.

### Predictor variable selection for nomograms

**Figure 2:**
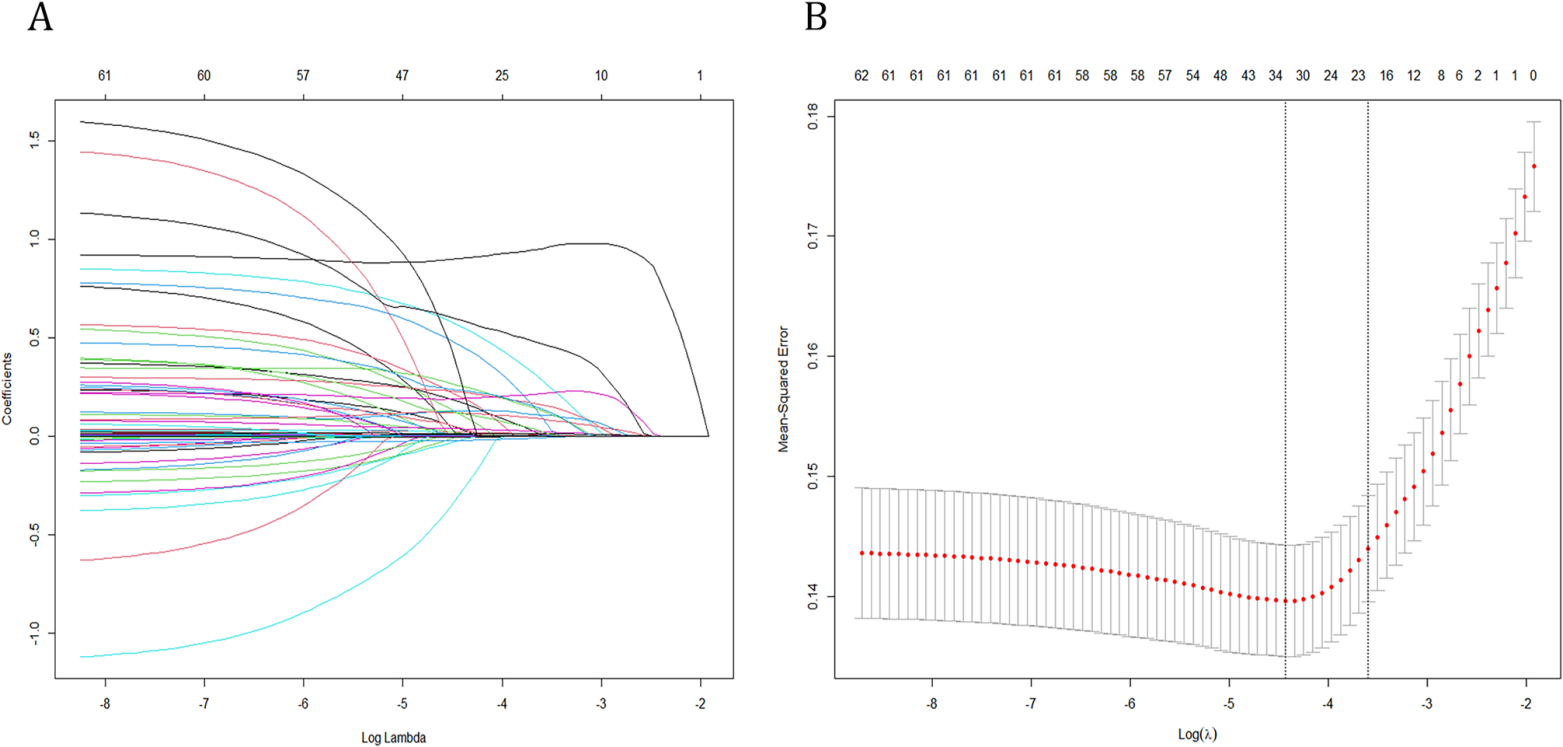
Predictor selection using the LASSO binary logistic regression model. (**A**) Tuning parameter (λ) selection using LASSO penalized logistic regression with tenfold cross-validation. (**B**) A minimum criteria and a 1-SE criteria were used to draw the dotted vertical lines representing the optimal values. A lambda value of 0.027378 was chosen (1-SE criteria).

LASSO regression was performed on 56 candidates (SOFA, MELD-Na and ABIC were not included), which included 21 continuous variables (Fig. 2). Twenty-one variables were suggested by LASSO regression to be associated with in-hospital mortality, including age, HE, sepsis shock, heartrate, MBP, WBC, MCV, TBIL, glucose, sodium, AG, PT, INR, blood infection, lung infection, fungal infections, carbapenem antibiotic, CRRT, mechanical ventilation, norepinephrine and vasopressin, which were included in the full model. The screening results are shown in Table 2.

**Table 2:** Results of multivariate logistic regression models to assess predictors of mortality for patients with liver cirrhosis and sepsis.

Variables	Full model			Simplified model		
	OR	95% CI	P-value	OR	95% CI	P-value
Age	1.037	1.023–1.051	< 0.001	1.039	1.024–1.053	< 0.001
HE	1.371	0.935–2.009	0.106			
Septic shock	1.363	0.896–2.073	0.147			
Heartrate	1.011	1.003–1.019	0.003	1.012	1.006–1.018	< 0.001
MBP	0.994	0.988–1.000	0.088			
WBC	1.012	0.992–1.032	0.240			
MCV	1.012	0.995–1.030	0.193			
TBIL	1.002	1.000–1.004	< 0.001	1.003	1.001–1.005	< 0.001
Glucose	0.997	0.995–0.999	0.011	0.996	0.994–0.998	0.001
Sodium	0.963	0.941–0.986	0.003	0.956	0.934–0.979	< 0.001
AG	1.049	1.012–1.086	0.007	1.062	1.029–1.096	< 0.001
PT	1.019	0.942–1.102	0.645			
INR	1.098	0.465–2.592	0.831			
Blood infection	1.244	0.778–1.987	0.362			
Lung infection	1.433	0.924–2.222	0.109			
Fungal infections	1.978	1.209–3.235	0.007	2.819	1.879–4.229	< 0.001
Carbapenem antibiotic	1.424	0.850–2.384	0.179			
CRRT	1.167	0.722–1.886	0.530			
Mechanical ventilation	2.252	1.580–3.211	< 0.001	2.326	1.670–3.239	< 0.001
Norepinephrine	1.211	0.795–1.846	0.374			
Vasopressin	2.500	1.571–3.978	< 0.001	3.912	2.675–5.722	< 0.001

*OR* odds ratio, *CI* confidence interval, *HE* hepatic encephalopathy, *MBP* mean blood pressure, *WBC* white blood cells, *MCV* mean red blood cell volume, *TBIL* total bilirubin, *AG* Anion gap, *PT* prothrombin time, *INR* international normalized ratio, *CRRT* continuous renal replacement therapy.

**Figure 3:**
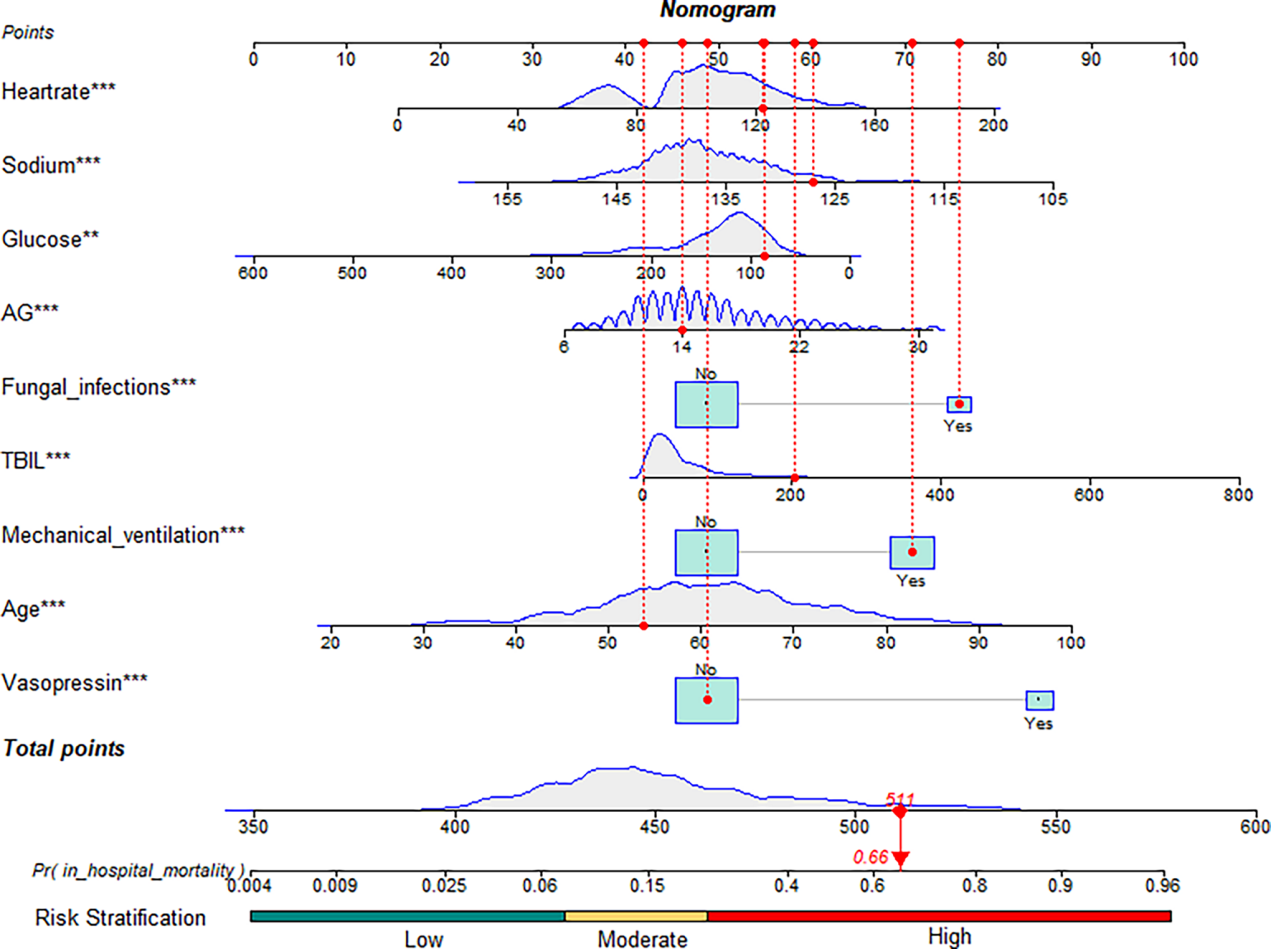
Nomogram for predicting hospital mortality among patients with liver cirrhosis and sepsis. *TBIL* total bilirubin, *AG* anion gap. **P < 0.01; ***P < 0.001.

In the multiple regression analysis, all nine variables screened were significantly associated with in-hospital mortality in patients with liver cirrhosis and sepsis (*P* < 0.05). The risk of in-hospital mortality was 3.912 times higher in patients receiving vasopressors (OR = 3.912, 95% CI 2.675–5.722). In-hospital mortality was 2.819 times higher in patients with fungal infections (OR = 2.819, 95% CI 1.879–4.229). In-hospital mortality was 2.326 times higher in patients on mechanical ventilation (OR = 2.326, 95% CI 1.670–3.239). Age (OR = 1.039, 95% CI 1.024–1.053), heartrate (OR = 1.012, 95% CI 1.006–1.018), TBIL (OR = 1.003, 95% CI 1.001–1.005), and AG (OR = 1.062, 95% CI 1.029–1.096) were risk factors for in-hospital death, while glucose (OR = 0.996, 95% CI 0.994–0.998) and sodium (OR = 0.956, 95% CI 0.934–0.979) were protective factors. A simplified model based on the above nine variables was developed and nomogram were drawn to predict the probability of in-hospital mortality in patients with liver cirrhosis and sepsis (Fig. 3). We additionally plotted risk stratification based on the tertiles of the total points: low risk (total points ≤ 437), moderate risk (437 < total points ≤ 463), and high risk (total points > 463). We also construct a dynamic nomogram (https://linhirog.shinyapps.io/Nomogram/) to assist in the application of the model.

### Nomogram verification

**Figure 4:**
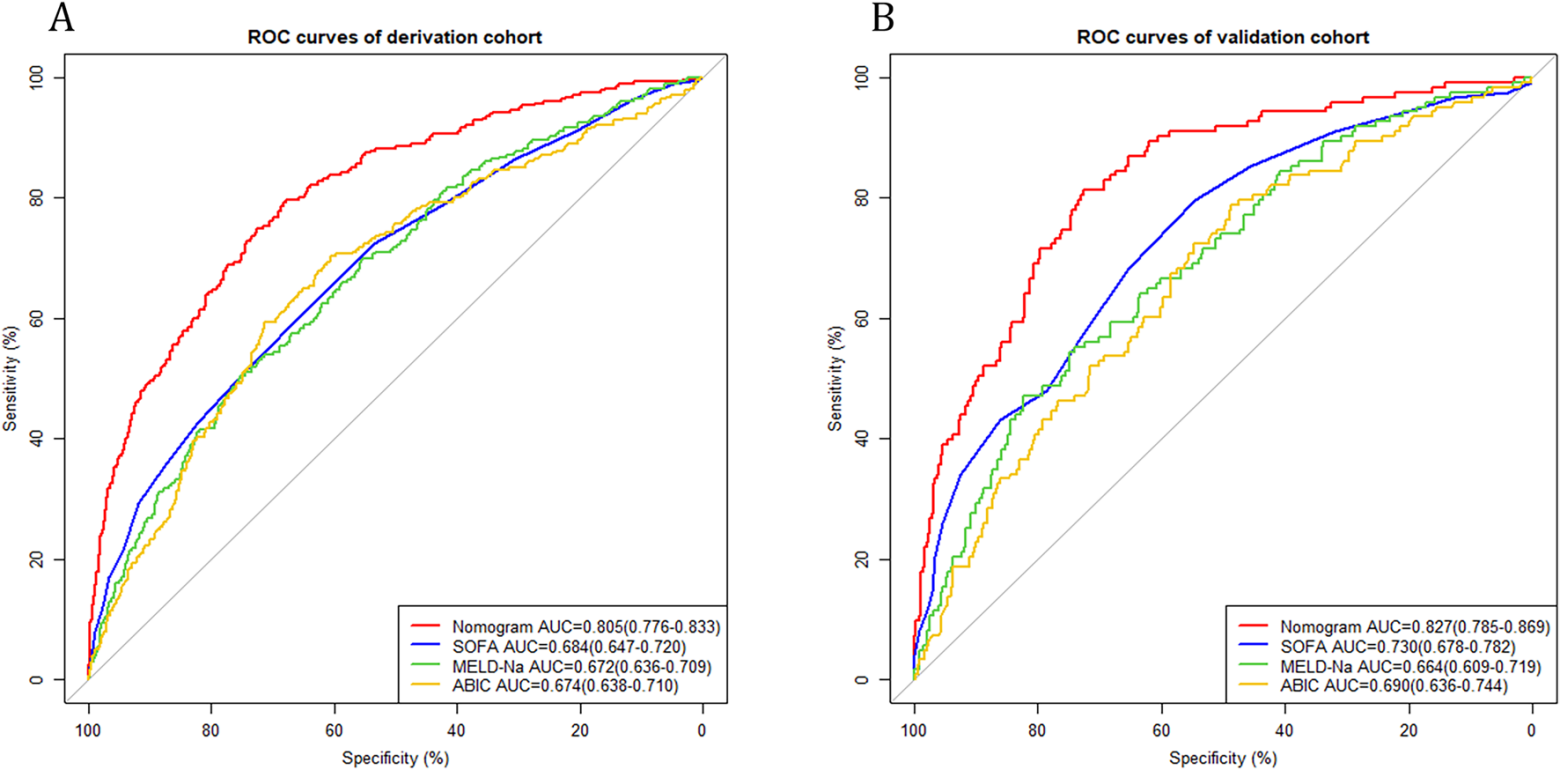
ROC curves. ROC curves were generated to validate the discrimination of the models, by the areas under the ROC curves. (**A**) ROC curves of the derivation cohort. (**B**) ROC curves of validation cohort. *SOFA* the sequential organ failure assessment, *MELD-Na* the model for end-stage liver disease with the incorporation of serum sodium, *ABIC* age-bilirubin-INR-creatinine score.

We calculated the AUC of the nomogram and compared the predictive performance of the nomogram, SOFA, MELD and ABIC score for in-hospital mortality in liver cirrhosis and sepsis. The results are listed in Table 3. The AUC values of the nomogram were0.805 (95% CI = 0.776–0.833) in the derivation cohort and 0.827 (95% CI 0.785–0.869) in the validation cohort, which were significantly higher than those of SOFA, MELD-Na and ABIC systems.The ROC curves are shown in Fig. 4. The corresponding IDI and NRI values also indicated that our nomograms had better discriminatory power and outperformed these previous prognostic prediction scoring systems.

**Table 3:** Comparison of models in predicting the in-hospital mortality of liver cirrhosis and sepsis patients.

Predictive model		AUC (95%CI)	*P*-value	IDI (95% CI)	P-value	NRI (95% CI)	P-value
Derivation cohort	Nomogram	0.805 (0.776–0.833)					
	SOFA	0.684 (0.647–0.720)	< 0.001	0.155 (0.127–0.183)	< 0.001	0.181 (0.119–0.244)	< 0.001
	MELD-Na	0.672 (0.636–0.709)	< 0.001	0.180 (0.149–0.210)	< 0.001	0.277 (0.216–0.339)	< 0.001
	ABIC	0.674 (0.638–0.710)	< 0.001	0.193 (0.163–0.223)	< 0.001	0.248 (0.187–0.309)	< 0.001
Validation cohort	Nomogram	0.827 (0.785–0.869)					
	SOFA	0.730 (0.678–0.782)	< 0.001	0.160 (0.105–0.215)	< 0.001	0.182 (0.070–0.294)	0.001
	MELD-Na	0.664 (0.609–0.719)	< 0.001	0.241 (0.187–0.295)	< 0.001	0.358 (0.250–0.466)	< 0.001
	ABIC	0.690 (0.636–0.744)	< 0.001	0.237 (0.184–0.289)	< 0.001	0.301 (0.197–0.405)	< 0.001

*AUC* the area under the receiver operating characteristic curve, *NRI* net reclassification index, *IDI* integrated discrimination improvement, *SOFA* the sequential organ failure assessment, *MELD-Na* the model for end-stage liver disease with the incorporation of serum sodium, *ABIC* age-bilirubin-INR-creatinine score.

**Figure 5:**
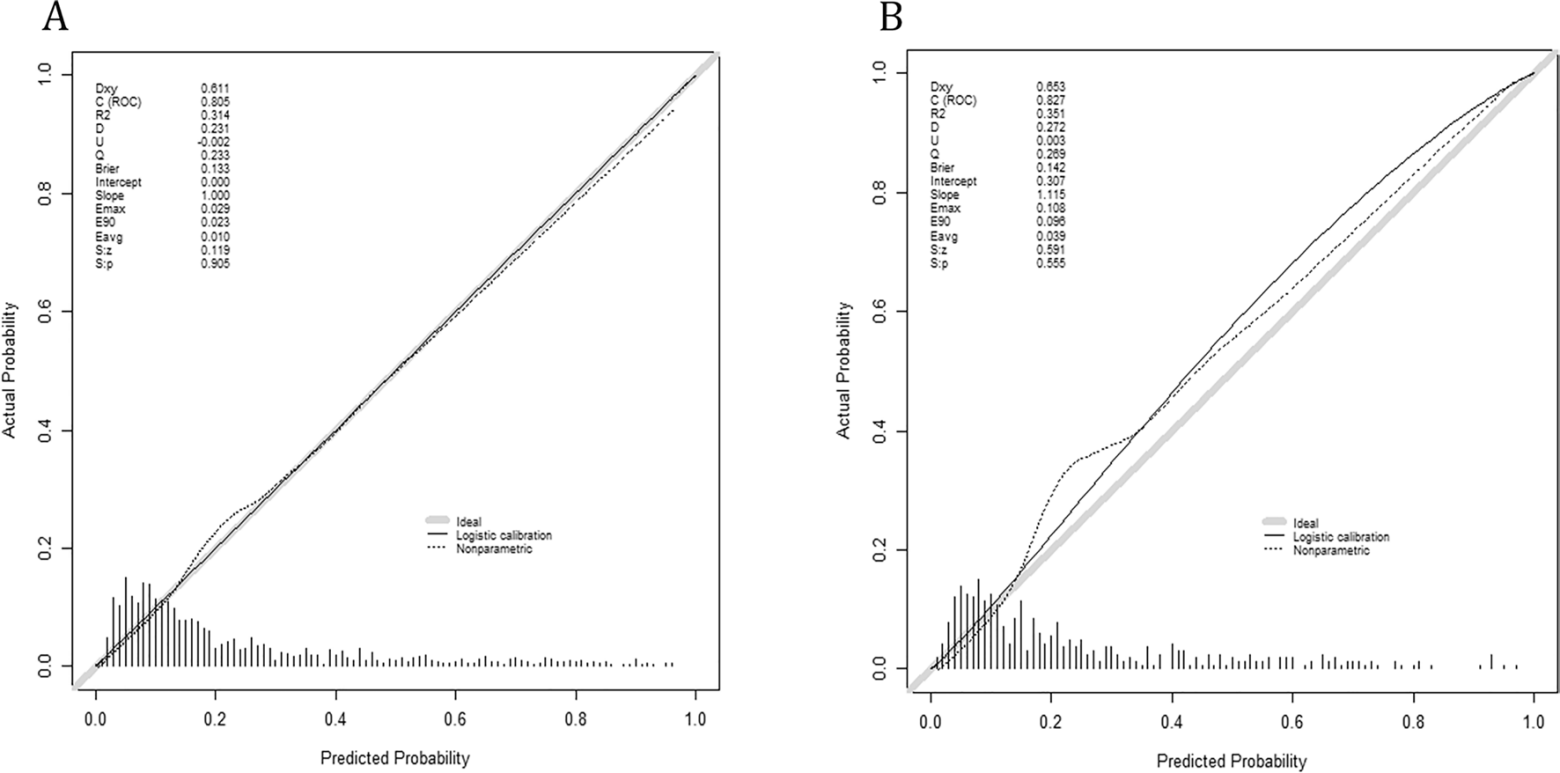
Calibration curves. Calibration curves depict the calibration of the newly established nomogram in terms of the agreement between the predicted probabilities and observed frequencies of the derivation cohort (**A**), and validation cohort (**B**).

The calibration curves showed that the column line plots for both cohorts were close to the diagonal, indicating a good fit (Fig. 5). In addition, the HL test demonstrated perfect agreement between predicted and observed values (derivation cohort, χ^2^ = 5.462, p = 0.792; validation cohort, χ^2^ = 11.879, p = 0.220). This indicates good agreement between predicted and observed values.

**Figure 6:**
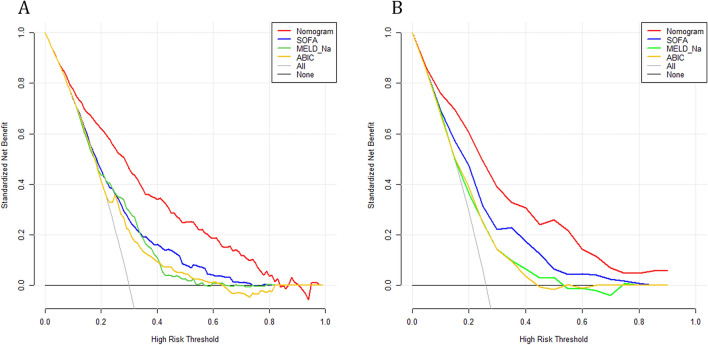
DCA curves of the derivation cohort (**A**), and validation cohort (**B**). *SOFA* the sequential organ failure assessment, *MELD-Na* the model for end-stage liver disease with the incorporation of serum sodium, *ABIC* age-bilirubin-INR-creatinine score.

We plotted DCA curves to illustrate the clinical value of columnar plots and compared them with the SOFA, MELD-Na and ABIC score (Fig. 6). The horizontal axis indicates that no one received the intervention and the net benefit was 0. The slanted line indicates that everyone received the intervention. Clinical interventions guided by columnar maps had greater net benefit than other scoring systems when the predicted probability thresholds in the development and validation cohorts were set at 10–80% and 10–90%.

## Discussion

Patients with cirrhosis are not only susceptible to acquiring infections due to immunodeficiency, but patients are frequently hospitalized and may carry underlying disease-specific susceptibilities, such as malnutrition or alcohol use disorders, which further increase the risk of infection^12^. Once sepsis occurs in patients with liver cirrhosis there is a significant increase in short and long-term mortality^13^. Also in-hospital mortality is significantly higher [27.7% (26.3–29.1%) vs. 3.7% (3.6–3.8%)]^14^. Although more studies exist on sepsis in the past, there is still a lack of research on sepsis in patients with cirrhosis^15^.

In our study, we used LASSO regression analysis to identify independent risk factors for in-hospital mortality in patients with liver cirrhosis and sepsis in MIMIC-IV. Development and validation of a convenient and effective nomogram to predict the prognosis of patients with cirrhosis and sepsis. As far as we know, this is the first nomogram for the prognosis of liver cirrhosis and sepsis. Our model showed similar performance not only in the derivation cohort but also in the validation cohort. Also, the nomograms showed good discriminatory power, calibration ability and clinical usefulness compared to SOFA, MELD-Na and ABIC score.

In our study, the incidence of septic shock in patients with cirrhosis and sepsis was 21.9%. LASSO regression analysis showed septic shock may be associated with patients with cirrhosis combined with sepsis. Norepinephrine, vasopressin, and terlipressin are commonly used vasoactive drugs in sepsis shock. The analysis of our study showed that norepinephrine and vasopressin were associated with in-hospital mortality in patients with cirrhosis and sepsis. And vasopressin was included in the final simplified model. Noteworthy, of the nine variables, vasopressin had the highest weight in the simplified model (OR = 3.912, 95% CI 2.675–5.722). Vasopressin is required for the occurrence of septic shock or a decrease in mean arterial pressure, yet it does not reduce the risk of in-hospital death in patients with cirrhosis and sepsis but increases it. We hypothesize that this may be related to the more severe degree of shock in patients using vasopressin and the fact that vasopressin further contributes to organ ischemia, and tachycardia, etc.^16^.

Our study showed a 12.4% prevalence of fungal infections in patients with liver cirrhosis and sepsis. Studies have indicated that fungal infections usually complicate the clinical course of patients with liver cirrhosis, leading to increased short-term mortality^17^. Our multivariate logistic regression analysis showed that the occurrence of fungal infections would increase in-hospital mortality by 2.819-fold. The occurrence of fungal infections is mostly hospital-acquired, mainly in the ICU^17,18^. This may be related to the fact that such patients are immunocompromised and receive more broad-spectrum antibiotics, invasive procedures, etc.

Bilirubin, serum sodium and serum AG were included in the final model. Elevated bilirubin levels occur not only in liver cirrhosis, but also in critically ill patients with hepatocellular damage due to hypoxic injury (ischemic cholestasis), and hyperbilirubinemia due to sepsis-associated cholestasis^19,20^. Studies have shown that hyperbilirubinemia is associated with poor outcomes in liver cirrhosis^21^ as well as in critically ill patients^22^. Patients with liver cirrhosis have impaired solute-free water excretion and dilutional hyponatremia due to nonosmotic secretion of antidiuretic hormone, activation of the renin–angiotensin–aldosterone axis and sympathetic nervous system^23^. Hyponatremia is a common problem of water-electrolyte disturbance in patients with advanced liver cirrhosis^24^. Our study showed that serum sodium concentration is a protective factor against in-hospital mortality in patients with liver cirrhosis and sepsis, and that lower blood sodium levels associated with inferior prognosis in patients with liver cirrhosis and sepsis. Interestingly, sodium is included in the formula for AG, and an increase in serum sodium would also indirectly reflect an increase in AG. However, increased AG is an independent risk factor for in-hospital mortality. AG is influenced by more indicators, the most common one leading to increased AG is metabolic acidosis, which implies the overproduction of organic acids, such as the accumulation of lactic acid^25^. It is hypothesized that this is related to an excess production of lactic acid due to hypoxia and shock caused by infection and a decrease in lactic acid clearance due to impaired hepatic clearance of lactic acid^26^. Among our studies, aging was associated with increased risk in patients with liver cirrhosis and sepsis. Also the use of mechanical ventilation is a factor that affects the prognosis of patients with liver cirrhosis and sepsis, and patients in the non-survival group are more prone to respiratory dysfunction due to pulmonary infections and ARDS and are often more critically ill, which may lead to a higher risk of death.

It was previously believed that sepsis patients have insulin resistance as well as an inflammatory response releasing inflammatory mediators that activate the neuro-endocrine system, causing an increase in blood glucose levels and leading to an increase in all-cause mortality in the ICU^27^. However, our study found that glucose was a protective factor for in-hospital mortality in patients with cirrhosis combined with sepsis (OR = 0.996, 95% CI 0.994–0.998). This may be related to the fact that patients with cirrhosis have impaired liver function and insufficient hepatic glycogen reserve, which makes them prone to hypoglycemia^28^. Appropriate maintenance of a certain level of blood glucose helps to provide the body with the necessary energy support and reduces the metabolic burden on the liver, thus exerting a protective effect on such patients.

This study also has some limitations: firstly since this is information from a single-center database and some variables such as lactate were not included in the study due to more than 20% of missing cases, as well as the unavailability of Child–Pugh score, both lead to a degree of bias. However, we set strict inclusion criteria together with exclusion criteria so that both the survivor and non-survivor groups would accurately reflect the true numbers. Secondly, our study was based on a retrospective cohort, and therefore the nomograms require further prospective validation before clinical application can be considered. Finally, the nature of observational studies suggests that unknown confounding factors may affect our results. Nomogram obviously do not provide fully accurate prognostic predictions and should therefore only be used by clinicians as a reference.

## Conclusion

We established the first prognostic nomogram based on the MIMIC database to predict in-hospital mortality in patients with liver cirrhosis and sepsis. In this retrospective cohort analysis of patients with liver cirrhosis and sepsis, we used LASSO logistic regression to identify 9 independent variables associated with in-hospital mortality, including age, heartrate,TBIL, glucose, sodium, AG, fungal infectiongs, mechanical ventilation and vasopressin. Based on a simplified model with 9 variables, nomogram were drawn to predict the risk of in-hospital mortality. The nomogram model performed well compared to SOFA, MELD and ABIC. The model performed well in internal validation and can be used to guide clinical practice, but further external prospective validation is needed.

## Data Availability

The data that support the findings of this study are available from the corresponding author upon reasonable request.
